# Prevalence, definition, and etiology of cesarean scar defect and treatment of cesarean scar disorder: A narrative review

**DOI:** 10.1002/rmb2.12532

**Published:** 2023-08-09

**Authors:** Shunichiro Tsuji, Yuri Nobuta, Tetsuro Hanada, Aike Takebayashi, Ayako Inatomi, Akimasa Takahashi, Tsukuru Amano, Takashi Murakami

**Affiliations:** ^1^ Department of Obstetrics and Gynecology Shiga University of Medical Science Otsu Shiga Japan

**Keywords:** cesarean scar defect, cesarean scar disorder, cesarean section, hysteroscopic surgery, secondary infertility

## Abstract

**Background:**

Cesarean scar defects (CSD) are caused by cesarean sections and cause various symptoms. Although there has been no previous consensus on the name of this condition for a long time, it has been named cesarean scar disorder (CSDi).

**Methods:**

This review summarizes the definition, prevalence, and etiology of CSD, as well as the pathophysiology and treatment of CSDi. We focused on surgical therapy and examined the effects and procedures of laparoscopy, hysteroscopy, and transvaginal surgery.

**Main findings:**

The definition of CSD was proposed as an anechoic lesion with a depth of at least 2 mm because of the varied prevalence, owing to the lack of consensus. CSD incidence depends on the number of times, procedure, and situation of cesarean sections. Histopathological findings in CSD are fibrosis and adenomyosis, and chronic inflammation in the uterine and pelvic cavities decreases fertility in women with CSDi. Although the surgical procedures are not standardized, laparoscopic, hysteroscopic, and transvaginal surgeries are effective.

**Conclusion:**

The cause and pathology of CSDi are becoming clear. However, there is variability in the prevalence and treatment strategies. Therefore, it is necessary to conduct further studies using the same definitions.

## Graphical abstract

The chronic inflammation that occurs in cesarean scar defect spreads into the uterine cavity and reduces fertility. Such secondary infertility has been termed cesarean scar disorder. This condition can be cured by endoscopic surgery.

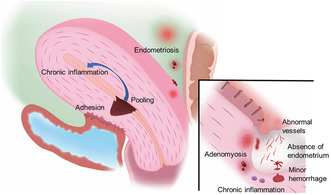

## 1 INTRODUCTION

Cesarean section (CS) is an effective and essential obstetric procedure that saves the lives of the mother and fetus, and its frequency is increasing worldwide. Although the World Health Organization has argued that CS rate should stay around 10%, the current global CS rate is 21.7%, almost doubling since 2000.^1^, ^2^, ^3^ The CS rate in Japan was approximately 19%.^4^ As the CS rate increases, concerns regarding its long‐term complications are also growing.

In recent decades, many studies have been conducted on cesarean scar defects (CSD), which may lead to gynecological symptoms, such as abnormal uterine bleeding (AUB), spotting, dysmenorrhea, chronic pelvic pain, dyspareunia, and secondary infertility.^5^, ^6^, ^7^, ^8^, ^9^, ^10^, ^11^, ^12^ Furthermore, they showed that the most common descriptor of CSD‐associated AUB was “brown discharge.” Additionally, CSD is described by other terms in the literature, such as isthmocele,^7^, ^13^, ^14^, ^15^ cesarean scar dehiscence,^16^, ^17^ uterine diverticulum,^18^, ^19^ niche,^20^, ^21^, ^22^ uterine transmural hernia,^23^ and cesarean scar pouch.^24^, ^25^ Although many terms express the lack of the myometrium in the anterior wall of the uterine isthmus due to CS, they are collectively referred to as CSD in this review.

We used the search terms ‘cesarean scar defect,’ ‘cesarean scar syndrome,’ ‘isthmocele,’ and ‘uterine niche’ to find and collect articles the PubMed database for articles published in the PubMed database up to December 2022. In this review, we present the definition, frequency, and etiology of CSD and clarify the pathophysiology and surgical treatment of symptomatic CSD. Additionally, we propose a treatment algorithm for secondary infertility in women with symptomatic CSD.

## 2 PREVALENCE AND DEFINITION OF CSD

According to recent review articles on CSD, the prevalence of CSD ranges between 24 and 70% using transvaginal ultrasound (TVS) and 56 and 84% using contrast‐enhanced sonohysterography.^8^, ^26^, ^27^ Because there are various definitions of CSD, it is not surprising that there are variations in its prevalence. De Vaate et al. defined CSD when the depth was >1 mm,^10^ and van der Voet et al. defined a CSD as an anechoic space (with or without fluid) at least 2 mm deep in the CS scar.^9^, ^28^, ^29^ Hayakawa et al. defined a wedge defect as a concavity with a depth of >5 mm.^30^ Other researchers have studied CSD characterized by a hypoechoic lesion in the anterior wall of the lower uterine segment without a clear definition.^6^, ^19^, ^24^, ^31^, ^32^ As a result, Jordans et al. defined the criteria of CSD as a depth of at least 2 mm through a consensus using the modified Delphi procedure involving 15 gynecological experts who were members of the European niche taskforce.^33^ CSD size is also important because as the CSD increases, AUB and uterine rupture during labor trials after CS also become frequent.^34^, ^35^

Furthermore, it is important to evaluate the timing of the menstrual cycle. Our previous report revealed that the prevalence of AUB in women with cesarean scar syndrome was during the follicular (48%) and ovulatory (42%) phases of the menstrual cycle, in contrast to the luteal phase (12%).^11^ Therefore, during the mid‐follicular phase, fluid collection in the CSD is more frequent than that of the luteal phase. This indicates that CSD may be overlooked on TVS examination if observed during the luteal phase.

TVS is the most commonly used technique for diagnosis.^11^ The definition of CSD is based on the value measured by TVS.^33^ On the other hand, several studies were conducted by magnetic resonance imaging (MRI) or hysteroscopy.^36^, ^37^, ^38^ MRI can uniformly evaluate the size and shape of CSD and the whole of the pelvis; however, TVS is limited by acoustic shadow and inter‐rater reliability. Hysteroscopy can provide information on CSD directly, such as the surface of CSD and the presence of an endometrium or a polyp. Therefore, it makes sense to use either MRI or hysteroscopy for research. However, in our study, TVS was deemed enough as the minimal information required for diagnosis, which is the depth of CSD, would be available with this technique. Furthermore, diagnosis using TVS makes economic sense in routine clinical practice.

## 3 ETIOLOGY OF CSD

### 3.1 Multiple CS

Many papers demonstrated that multiple CS was a major risk of CSD.^6^, ^27^, ^28^, ^39^ Osser et al. also demonstrated that myometrial thickness at the level of the isthmus was 8.3, 6.7, and 4.7 mm in women who had one, two, and at least three CS procedures, respectively, although it was 11.6 mm in women who had only vaginal delivery.^39^ Cesarian scar defect was detected in 61%, 81%, and 100% of women who underwent one, two, and three CS procedures, respectively.^39^ In another prospective cohort study, CSD was found to have developed in 35%, 63%, 76%, and 88% of women with 0, 1, 2, and 3 CS, respectively.^28^

### 3.2 Cervical dilatation

Active labor and cervical dilatation were significant risk factors for CSD, especially cervical dilatation >5 cm.^13^, ^26^, ^27^ Vikhareva et al. showed that the odds ratio of a large CSD compared to 0 cm cervical dilatation was 4.4 (95% CI, 0.7–28.5), 26.5 (95% CI, 4.3–161.8), and 32.4 (95% CI 6.1–171.0) at 1–4, 5–7, and 8 cm or more cervical dilatation, respectively.^40^ If the station of the presenting part of the fetus at CS was at the pelvic inlet, it was also a risk factor for a large CSD compared to that below the pelvic inlet.^40^ Emergency CS also contributed to CSD formation when compared to elective CS.^41^ However, some studies have shown that emergency CS was not an independent risk factor for CSD.^28^, ^30^

### 3.3 Uterine closure technique

The single‐layer closure technique for the myometrium during CS is associated with a higher risk of CSD than the double‐layer closure procedure.^26^, ^30^, ^42^ Roberge et al. also showed that double‐layer closure with an unlocked first layer was a significant association with thicker residual myometrial thickness (RMT); furthermore, if a double‐layer closure was conducted with a locked first layer, there was no significant difference compared with single‐layer closure in either RMT.^43^ Ceci et al. demonstrated that continuous single‐layer closure contributed to a larger defect compared with interrupted sutures.^44^ This has been described in the Discussion section of their paper as follows: the ischemic condition of the myometrium negatively affected the healing of cesarean scars.^44^ In a systematic review and meta‐analysis, Qayum et al. concluded that a double‐layer closure contributed to a thicker RMT and lower incidence of dysmenorrhea than a single‐layer closure.^45^

### 3.4 Level of uterine incision

Vikhareva et al. suggested that a low level of hysterectomy during CS is more frequently associated with CSD development and that cervical tissue attenuates the healing of cesarean scars when included in the closing tissue.^40^ They also demonstrated that a low hysterectomy level during CS was significantly associated with a higher incidence of large CSD in a randomized, single‐blinded trial.^35^ Additionally, Vervoort et al. described low incisions through cervical tissue, including mucus‐producing glands, which prevented the healing of the cesarean wound.^46^

### 3.5 Pelvic adhesion

Adhesion of the vesicouterine pouch 18 months after CS was an independent risk factor for CSD according to the results of the univariate logistic regression analysis.^41^ Vervoort et al. also described that counteracting forces on the uterine scar by adhesion formation between the uterine scar and the abdominal wall might impair wound healing and develop the formation of CSD.^46^

### 3.6 Obstetrical complication

Hayakawa et al. explored the risk of CSD among perioperative parameters in a prospective study. According to the multivariate logistic regression analysis in that study, multiple pregnancies, premature rupture of membranes, and pre‐eclampsia were significantly linked to CSD formation (OR, 8.94; 95% CI, 1.97–40.61; OR, 8.72; 95% CI, 1.28–59.65; and OR, 8.71; 95% CI, 1.70–44.54, respectively).

As described above, CSD is considered to occur depending on the situation and procedure at the time of CS. Although CSD cannot be avoided in some cases, an iatrogenic component might be associated with the development of CSD. Therefore, in the future, it will be desirable to establish a valid CS method to prevent CSDi development.

## 4 SYMPTOMS ASSOCIATED WITH CSD AND ITS NOMENCLATURE

Abnormal genital spotting was observed in approximately 30% of women with CSD at 6–12 months after CS.^46^ A prospective study by van der Voet et al. showed the relationship between CSD and postmenstrual spotting.^9^, ^39^ According to this report, 13 (28.9%) of 45 women with CSD, detected using gel instillation sonography (GIS) at 12 months after CS, had postmenstrual spotting. In contrast, in 29 women without CSD, only 2 (6.9%) had postmenstrual spotting (odds ratio [OR], 5.48; 95% confidence interval [CI], 1.14–26.48). When a large CSD was classified as a residual myometrial thickness (RMT) of <50% of the adjacent myometrium detected by GIS like Ofili‐Yebovi et al.,^47^ women with large CSD had postmenstrual spotting more frequently than women without large CSD (OR, 6.1; 95% CI, 1.94–26.70).^9^ A recent systematic review and meta‐analysis by Murji et al.^34^ also demonstrated a strong and consistent association between patients with CSD and AUB. Furthermore, they showed that the most common descriptor of CSD‐associated AUB was “brown discharge.”

Wang et al. showed that the prevalence of postmenstrual spotting in 207 women with CSD, dysmenorrhea, chronic pelvic pain, and dyspareunia was 63.8%, 53.1%, 39.6%, and 18.3%, respectively.^6^ A combination of these symptoms was unique. Our previous study showed that women with dysmenorrhea were included in women with AUB, and women with chronic pelvic pain were included in women with dysmenorrhea.^11^ These events indicate that there is an order in which symptoms appear and that this condition is progressive.

In a recent prospective study, Dosedla et al. demonstrated that 4 (40%) of 10 women with severe CSD complained of dysmenorrhea; in contrast, 14 (7.4%) of 190 women with normal CS scars complained of dysmenorrhea at 18 months after CS. Three (30%) women had severe CSD, although 14 (7.4%) complained about chronic pelvic pain. These results indicate a significant association between CSD and these symptoms.^41^

Isolated or combined symptoms are expressed as niche‐related symptoms or symptomatic isthmoceles.^48^, ^49^ Morris advocated calling these symptoms cesarean scar syndrome.^50^ Since the term describing this condition was not unified, a task force was established to avoid confusion under these conditions.^51^ This task force, which comprised experts with experience in the field, provided results using the electronic Delphi method. Therefore, this condition was termed cesarean scar disorder (CSDi). This task force also defined the diagnostic definition of CSDi as a consensus among experts. However, some points must be considered during the diagnosis. First, the cause of AUB should be ruled out according to the International Federation of Gynecology and Obstetrics classification system (PALM‐COEIN).^51^ Symptoms must be confirmed in at least three menstrual cycles before diagnosis. These symptoms also need to develop and worsen after CS to qualify for CSDi. Based on this study, this review refers to this condition as CSDi.

## 5 HISTOPATHOLOGY OF CSD IN WOMEN WITH CSDI

Morris first explored the microscopic findings of CSD.^52^ This report showed congested endometrium above a scar (61%), polyp formation (16%), lymphocytic infiltration (65%), residual suture material (92%), capillary dilatation (65%), free red blood cells in the endometrial stroma of the scar (59%), fragmentation and breakdown of the endometrium of the scar (37%), and adenomyosis confined to the scar (28%) in a series of 51 hysterectomy specimens. However, these findings were obtained from patients who did not desire fertility. Donnez demonstrated that muscle fiber density was significantly lower compared with adjacent myometrium and the presence of endometriosis (21%) in 38 patients with CSDi who underwent laparoscopic repair of the defect.^53^ AbdullGaffar et al.^21^ reported histological findings of CSD obtained by hysterectomy and hysteroscopic resection. Fibromuscular stroma with thick blood vessels was observed in all cases. They also described differences between hysteroscopically resected CSD and hysterectomy specimens. Although there was obscured or unidentified luminal and mucosal hemorrhage in hysteroscopic isthmocele, 56% of patients had luminal hemorrhagic debris or extravasated red blood cells in the mucosal stroma of CSD.^21^ Regarding hemorrhage from CSD, Tanimura et al. also showed using hysteroscopy that hemorrhage occurs from CSD.^54^ Our group studied histopathological findings of issues obtained from CSD lesions in women who underwent laparoscopic resection due to CSDi.^55^ This study demonstrated that the proportion of endometrium present on the surface of CSD was lower in women with CSDi than in women with non‐CSDi (22% vs. 62%, respectively). The presence of adenomyosis was also detected (43%) in CSD lesions among women with CSDi. Furthermore, immunohistochemical analysis indicated that chronic inflammation occurred in CSD. These studies suggest that there is a lack of healthy and normal endometrium on the surface of CSD in women with CSDi. Instead, fibrotic tissue with thick blood vessels was observed. Furthermore, minor hemorrhages and chronic inflammation often occur in CSD. Piriyev et al.^56^ also reported adenomyosis in 12 (42.9%) patients and fibrosis in 9 (32.1%) patients. Collectively, fibrosis and adenomyosis are considered the main histological findings of CSD.

## 6 DECREASED FERTILITY IN WOMEN WITH CSDI

Does CS affect subsequent fertility? Kjerulff et al.^57^ showed that the subsequent birth rate after CS was 15% lower than after vaginal birth. From the point of view of assisted reproductive technology (ART), Vissers et al.^58^ demonstrated that live birth rates were significantly lower in women with a previous CS than in women with a previous vaginal delivery in an analysis involving 1317 women. In a systematic review and meta‐analysis, Gurol‐Urganci, et al.^59^ revealed that CS had a significant negative effect on subsequent pregnancy rates (risk ratio, 0.91; 95% CI, 0.87–0.95) compared with vaginal delivery. In contrast, they also demonstrated different conclusions in a population‐based cohort study, in which CS had none or slightly negative effects on future fertility if the study was conducted only among low‐risk primiparous women.^60^ Although it seems inconsistent, it is reasonable based on the CSD risk. As mentioned above, high‐risk pregnancies are significantly associated with CSD. Therefore, in the low‐risk group, there was little impact on fertility following CS.

However, the pathophysiological reasons why CS leads to decreased fertility remain unclear. Florio et al. described that the persistence of blood in CSD deteriorated mucus and sperm quality, obstructed sperm passing through the cervix, and eventually affected the implantation of the embryo.^7^ According to the histopathology findings mentioned above, chronic inflammation can be considered as a cause. Hsu et al.^61^ reported that bacterial colonization is more frequent in women with secondary infertility due to CSD. Microorganisms may be present because of the retention of blood in CSD and contribute to chronic inflammation. Our group demonstrated that the frequency of chronic endometritis in women with CSDi was significantly higher than that in the general infertile population.^62^ Additionally, we showed that higher levels of inflammatory cytokines (tumor necrosis factor‐a and interleukin‐1b) in women with CSDi were detected in the uterine cavity compared to women with a history of CS but without symptoms of CSDi. Bi et al.^18^ showed, using correlation analysis, that levels of inflammatory factors (tumor necrosis factor‐a, interleukin‐1b, and interleukin‐6), size of uterine scar diverticulum, and myometrial thickness at uterine scar were significantly correlated with subsequent infertility. These chronic inflammatory conditions within the CSD and pelvic cavity can impair embryonic implantation. Florio et al.^7^ reported that the persistence of accumulated blood deteriorated mucus and sperm quality.

**FIGURE 1:**
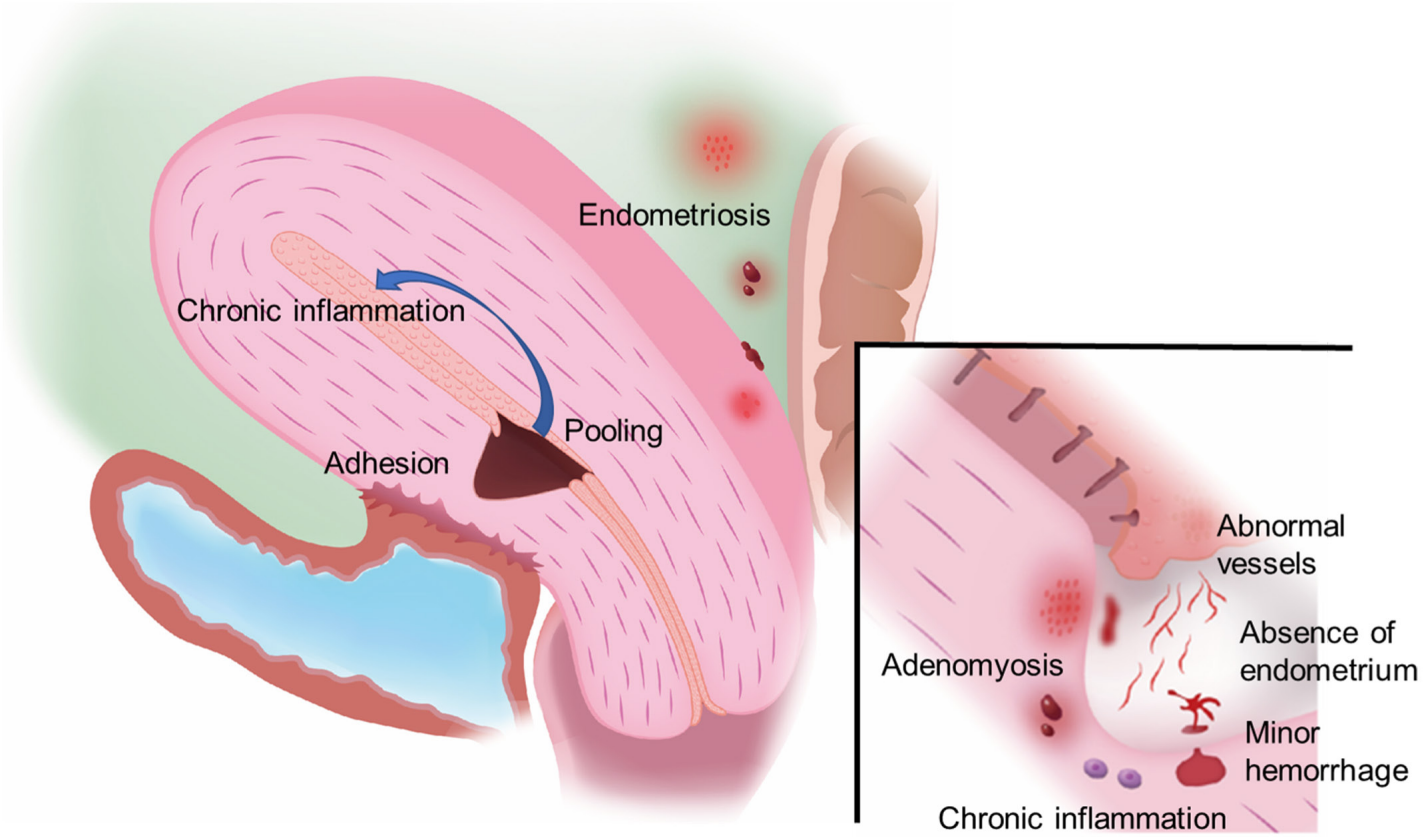
Schema representing the pathophysiology of cesarean scar disorder. The absence of endometrium and abnormal vascular development is visible on the surface of the cesarean scar defects (CSD). Occasionally, microhemorrhages can be directly observed in CSD. In the myometrium under CSD, ectopic endometrium and CD138‐positive plasma cells can be observed. Chronic inflammation generated in CSD may spread into the uterine cavity and induce chronic endometritis. Furthermore, endometriosis, a chronic inflammatory disease, is frequently found in the pelvis of patients with cesarean scar disorder (CSDi).

Dysmenorrhea and chronic pelvic pain are symptoms of CSDi experienced by women with endometriosis. Gulz et al.^49^ showed that endometriosis was diagnosed during laparoscopic surgery in 22 of 83 patients (27%). In contrast, our recent report demonstrated that endometriosis was detected in 51 (70%) patients with CSDi, although majority of patients were not at a severe stage of endometriosis.^62^ Of these patients with endometriosis, 85% had only peritoneal lesions. Endometriosis also causes infertility, owing to chronic inflammation. Yang et al.^63^ reported cystic adenomyosis associated with CSD. The cystic wall contained endometrium‐like tissue in the inner wall and chocolate‐like fluid inside. These findings suggest that CSDi is associated with endometriosis. Overall, these results suggest that multiple factors negatively affect fertility in women with CSDi (Figure 1).

## 7 PHARMACOLOGICAL TREATMENT FOR AUB AND DYSMENORRHEA IN WOMEN WITH CSDI

Gynecological symptoms, such as AUB and spotting between menstruations, can be treated with medical management, such as oral contraceptives or a levonorgestrel intrauterine system (LUS).^64^, ^65^, ^66^ However, there are not enough studies regarding pharmacological treatment for gynecological symptoms. Therefore, a study regarding the effectiveness of Chinese herbal medicine is underway.^67^ In addition, hormone therapy has an ovulation‐suppressing effect and cannot be used to treat secondary infertility. Therefore, it is necessary to consider the following ART and surgical therapy for the treatment of secondary infertility.

## 8 ART FOR SECONDARY INFERTILITY IN WOMEN WITH CSDI

Assisted reproductive technology has become an integral part of infertility treatment. Lawrenz et al.^68^ recommended paying attention to the presence of intracavity fluid (ICF) during ultrasound monitoring for ovarian stimulation; they suggested that eliminating ICF before embryo transfer might contribute to maintaining reproductive outcomes compared to patients without CSD. Huang et al.^69^ demonstrated that CSD adversely affects pregnancy and live birth rates after in vitro fertilization by comparing 215 patients with CSD and 1323 patients without CSD. Furthermore, their results highlighted that ICF decreased both pregnancy and live birth rates. Gurbuz et al. showed that the administration of the GnRH agonist, leuprolide acetate, for 3 months prior to the embryo transfer cycle was effective in women with CSDi. This method was inspired by an ultra‐long protocol in patients with endometriosis.^70^ Since the histopathology of CSD is characterized by endometriosis, their protocol is considered reasonable.^52^, ^55^, ^71^ Although ART can have some effects on infertile women with CSDi, our previous report demonstrated that the pregnancy rate following typical treatment, including ART for infertile women with CSDi, was inferior to operative treatment.^11^ Baldini et al.^72^ reported no association between CSD and cesarean scar pregnancy (CSP) in patients undergoing in vitro fertilization; however, day 5 transfer at the blastocyst stage might be superior to day 3 embryo transfer with regard to preventing CSP.

As mentioned above, there are several reports on ART treatment for women with CSDi, although the number of such reports is insufficient. To date, there are no reports of ART being superior to surgical therapy. Therefore, surgical therapy should be considered first, or in cases of no conception, even after the transfer of a good embryo.

## 9 LAPAROSCOPIC SURGERY IN WOMEN WITH CSDI

Laparoscopic repair of CSD in a patient with CSDi was first described by Jacobson et al.^73^ as a case report. However, the true pioneers of laparoscopic repair of CSD were Donnez et al.,^74^ whose group demonstrated three cases of laparoscopic repair and later published a series on the same.^53^, ^75^ Many authors then showed the effectiveness of laparoscopic repair.^65^, ^71^, ^76^, ^77^, ^78^, ^79^, ^80^, ^81^, ^82^, ^83^, ^84^, ^85^, ^86^, ^87^ According to previous reports, there are two points that we have to pay attention to in laparoscopic surgery for CSDi (Table 1).

**TABLE 1:** Laparoscopic surgery for CSDi.

Author	Year	No	Identification of CSD	Suture technique	Pre‐RMT	Post‐RMT	Effectiveness	Pregnancy rate
Donnez et al.	2008	3	Insert probe	Double layer	1.2 ± 0.5 mm	10.2 ± 0.3 mm	Hyper (100%) PP (100%)	33%
Marotta et al.	2013	13	Insert probe	Double layer	1.7 ± 0.7 mm	9.8 ± 1.0 mm	Hyper (100%) Dys (100%) PP (100%)	31%
Li et al.	2014	17	Insert probe	Single layer	2.4 ± 1.3 mm	9.5 ± 1.3 mm	AUB (92%), PP (100%)	66%
Masuda et al.	2015	1	Insert bipolar device	NA	NA	NA	AUB (100%)	100%
Tanimura et al.	2015	18	Hysteroscopy	Double layer	2.8 (range, 1–6.1) mm	10.5 (range, 6.8–14) mm	AUB (89%)	56%
Nirgianakis et al.	2016	21	Hysteroscopy	Single layer	NA	NA	NA	NA
Zhang Y.	2016	59	Hysteroscopy	Single layer	NA	NA	AUB (89%)	NA
Zhang X et al.	2016	86	Hysteroscopy	Double layer	NA	NA	AUB (significant improvement)	38%
Liu et al.	2016	49	Hysteroscopy	Double layer	NA	NA	AUB (90%)	NA
Bakavičiūtė et al.	2016	1	Hysteroscopy	Single layer	NA	NA	NA	100%
Aimi G. et al.	2017	1	Ultrasonography	Single layer	2.6 mm	NA	AUB (100%) PP (100%)	NA
Donnez O et al.	2017	38	Insert probe	Double layer	1.4 ± 0.7 mm	9.6 ± 1.8 mm	AUB Dys PP (91%)	44%
Dosedla E et al.	2017	11	Insert probe	Single layer	0.3 ± 0.4 mm	1.3 ± 1.0 mm	AUB (82%), Dys (82%), PP (73%)	NA
Vervoort et al.	2018	101	Hysteroscopy	Double layer	1.2 (IQR, 0.8–1.7) mm	5.3 (IQR, 4.0–6.9) mm	AUB, Dys, PP (SI)	NA
Lv et al.	2018	31	Hysteroscopy	Double layer	0.9 (0.1–3.9) mm	NA	AUB (80%)	62%
Zhang Y.	2020	45	Hysteroscopy	Only serosa side^a^	3.3 ± 2.7 mm	NA	AUB (SI)	42%
Karampelas	2021	31	NA	Double layer	1.8 ± 0.9 mm	6.7 ± 1.8 mm	AUB (71%), PP (83%)	83%
Zhang, N et al.	2021	40	Hysteroscopy	Only serosa side^a^	2.0 ± 0.4 mm	6.0 ± 0.8 mm	AUB (93%)	50%
		36	Hysteroscopy	Single layer	2.0 ± 0.4 mm	5.9 ± 1.0 mm	AUB (86%)	25%
Piriyev et al.	2022	28	Hysteroscopy	Single or Double	2.0 mm	8.7 mm	NA	NA
Goldenberg et al.	2022	48	NA	Double layer	2.0 (IQR, 1.4–2.5) mm	NA	AUB (75%)	81%
Peng et al.	2022	11	Hysteroscopy	Muscle flap filling suture	2.1 ± 1.4 mm	6.7 ± 1.8 mm	AUB (100%)	36%
		12	Hysteroscopy	Folding suture^a^	1.8 ± 0.9 mm	6.3 ± 1.7 mm	AUB (100%)	58%

Abbreviations: AUB, abnormal uterine bleeding; CSD, cesarean scar defect; CSDi, cesarean scar disorder; Dys, dysmenorrhea; Hyper, hypermenorrhea; IQR, interquartile range; NA, not available; No., The total number of cases in the study; PP, pelvic pain; RMT, residual myometrial thickness; SI, significant improvement; (%), effective rate.

^a^ Without incision of cesarean scar defect.

The first is how to identify CSD after opening the uterovesical peritoneum and separating the bladder from the uterus. Donnez et al.^74^ and Li et al.^76^ used a probe via the vaginal approach to detect CSD, whereas Dosedla et al.^81^ selected a uterine probe through the cervical canal into the uterine isthmus. Masuda et al.^77^ used a hysteroscopic bipolar device. However, most authors have detected CSD using hysteroscopic guidance.^56^, ^65^, ^71^, ^78^, ^79^, ^80^, ^82^, ^88^, ^89^, ^90^, ^91^, ^92^ Nirgianakis K et al.^79^ named the see‐through light from CSD by hysteroscopy as the Halloween sign, and called a combination of laparoscopy and hysteroscopy in the repair for CSD, the Rendezvous technique. Because the extent of CSD resection cannot be accurately identified in the peritoneal cavity, direct confirmation using hysteroscopy seems reasonable. Recently, Sako et al.^93^ reported a non‐perfusion hysteroscopic technique. Intraperitoneal gas was allowed to flow into the uterus through a CSD incision. Therefore, a hysteroscopic view can be obtained without using a hysteroscopic perfusion fluid. This report also proposes hysteroscopy to precisely identify lesions that should be resected.

The second point is how to suture the uterine myometrium after resection. Several authors performed double‐layer sutures,^53^, ^65^, ^71^, ^74^, ^75^, ^78^, ^82^, ^85^, ^90^, ^94^ whereas several authors performed single‐layer sutures.^76^, ^79^, ^80^, ^81^, ^89^, ^91^, ^95^ So far, no papers compare the superiority of single‐layer and double‐layer sutures for CSD repair. Recently, there have been a couple of reports on suturing only the uterine serosa without CSD excision. Zhang et al. demonstrated that the surface of the uterine serosa was condensed by vertical suturing without CSD removal.^88^ Zhang et al.^91^ also demonstrated that laparoscopic repair without scar resection is feasible, safe, and effective for AUB by comparing resection procedures. Peng et al.^92^ demonstrated that both folding and muscle flap filling suture techniques are effective and safe in women with CSDi.

Several reports of laparoscopic repair have recommended bilateral ligation of the round ligaments when the uterus is straight or retroflexed.^53^, ^71^, ^96^ This technique is thought to contribute to the reduction of CSD repair site tension and promote wound healing, which contributes to an increase in RMT.

A laparoscopic repair can dramatically increase RMT and relieve various symptoms. Based on the evidence, approximately 90% of symptom improvement can be expected. Regarding pregnancy rates, there was a wide range between 25 and 100%. Most reports indicate that the postoperative period of contraception is 3–6 months. To date, there have been no reports of uterine rupture in pregnancies following laparoscopic repair.

Based on the above, we believe that the optimal laparoscopic surgery at present is to use a hysteroscope in combination to excise the CSD accurately, suture the wound in two layers, and in some cases, correct the uterine position by suturing the round ligament.

## 10 HYSTEROSCOPIC SURGERY IN WOMEN WITH CSDI

Fernandez et al.^97^ first described hysteroscopic surgery for patients with CSDi. Two of four women with infertility conceived following hysteroscopic surgery. Thereafter, many studies have demonstrated the safety and effectiveness of this surgery (Table 2). Hysteroscopic surgery offers several advantages. The key points are resection and coagulation. Regarding the resected part, three methods were used: only the inferior edge, both the superior and inferior edges, and both the bipolar edge and bottom of the CSD. It seems that there was not much of a difference in these effects. In contrast, electrocauterization of the CSD surface is considered common. Recently, Casadio et al.^98^ showed a new technique for hysteroscopic surgery in which all walls of the isthmus were resected to completely remove fibrotic tissues, and electrocoagulation was performed on both the CSD surface and along the cervical canal walls. This radical excision and cauterization approach make sense from a pathophysiological perspective. Furthermore, this technique can contribute to increase in RMT, decrease the size of CSD and improve gynecological symptoms due to CSD. Shi et al.^99^ indicated the proper timing of hysteroscopic surgery for CSDi. The timing was within 14 days of menstruation and may contribute to relief from various symptoms of CSDi in women without intentions of childbearing. Huang et al^100^ also demonstrated the effectiveness of radical excision for patients with prolonged menstrual spotting.

**TABLE 2:** Hysteroscopic surgery for CSDi.

Author	Year	No	Resecting part	Electrocauterization area	Pre‐RMT	Post‐RMT	Effectiveness	Pregnancy rate
Fernandez et al.	1996	4	Inferior edge of CSD	NA	NA	NA	NA	50%
Fabres et al.	2005	24	Inferior edge of CSD	NA	NA	NA	AUB (84%)	45%
Gubbini et al.	2008	26	Bipolar edge of CSD and bottom of CSD	Bottom of CSD	NA	NA	AUB (100%)	78%
Chang et al.	2009	22	Inferior edge of CSD	Vessels on CSD	3.9 ± 1.9 mm	NA	AUB (64%)	NA
Flori et al.	2011	19	Bipolar edge and bottom of CSD	Bottom of CSD	NA	NA	AUB (100%), PP (63%)	NA
Wang et al.	2011	57	Inferior edge and bottom of CSD	Bottom of CSD	NA	NA	AUB (60%)	NA
Gubbini et al.	2011	41	Bipolar edge and bottom of CSD	Bottom of CSD	NA	NA	AUB (100%)	100%
Feng et al.	2012	62	Inferior edge of CSD	Bottom of CSD	NA	NA	AUB (61%)	NA
Li et al.	2014	24	Bipolar edge of CSD	Bottom of CSD	4.8 ± 1.6 mm	5.6 ± 1.7 mm	AUB (76%) PP (100%)	NA
Raimondo et al.	2015	120	Bipolar edge and bottom of CSD	NA	NA	NA	AUB (80%)	NA
Tanimura et al.	2015	4	Bipolar edge and bottom of CSD	Bottom of CSD	NA	NA	NA	100%
Zhang et al.	2016	19	Inferior edge of CSD	NA	NA	NA	AUB (SI)	NA
Muzii	2017	23	Inferior edge and bottom of CSD	Bottom of CSD	NA	NA	AUB (SI)	NA
Tsuji et al.	2018	18	Inferior edge of CSD	Bottom of CSD	2.8 (range, 0.8–5.4) mm	4.2 (range, 1.0–7.8) mm	NA	40%
Vervoot et al.	2018	45	Inferior edge of CSD	Bottom of CSD	4.0 (IQR, 3.4–6.0)	NA	AUB (SI)	NA
Lv et al.	2018	51	Bipolar edge and bottom of CSD	Bottom of CSD	1.0 (0.5–3.8)	NA	AUB (70%)	73%
Albornoz et al.	2019	38	Inferior edge of CSD	Bottom of CSD	NA	NA	AUB (97%), PP, Dys, Dysp (SI)	43%
Calzolari et al.	2019	35	Bipolar edge and bottom of CSD	Bottom of CSD	NA	NA	NA	56%
Shapira et al.	2020	67	Bipolar edge and bottom of CSD	NA	NA	NA	AUB (63%)	53%
Tsuji et al.	2020	38	Inferior edge of CSD	Bottom of CSD	2.2 (IQR, 1.1–3.7) mm	4.4 (IQR, 2.5–6.0) mm	NA	71%
Shi et al.	2020	124	Inferior edge of CSD	Bottom of CSD	4.9 ± 2.1 mm	5.1 mm (SI) 4.9 mm (NI)	AUB (66%)	NA
Zhu et al.	2020	208	Inferior edge of CSD	Bottom of CSD	3.4 ± 1.5 mm	NA	AUB (64%)	NA
Zeller et al.	2021	49	Whole surface of CSD	Whole surface of CSD	1.7 (IQR, 0.5–2.0) mm	NA	NA	74%
	2021	22	Whole surface of CSD	Whole surface of CSD	5.0 (IQR, 4.0–6.0) mm	NA	NA	64%
Nguyen	2022	23	Bipolar edge of CSD	Bottom of CSD	3.9 ± 0.38	4.0 ± 0.38	AUB (69%), PP (94%)	30%^a^
Casadio et al.	2022	32	Bipolar edge and channel‐like 360°	Bottom of CSD	2.3 ± 0.3 mm	4.3 ± 0.7 mm	AUB (97%), PP, Dys, Dysp (SI)	NA
Huang et al.	2022	66	Bipolar edge of CSD	Bipolar edge and bottom of CSD	NA	NA	AUB (98%)	NA

Abbreviations: AUB, abnormal uterine bleeding; CSD, cesarean scar defect; CSDi, cesarean scar disorder; Dys, dysmenorrhea; Dysp, dyspareunia; IQR, interquartile range; NA, not available; NI, no improvement; PP, pelvic pain; SI, significant improvement; (%), effective rate.

^a^ Within 6 months after surgery.

**FIGURE 2:**
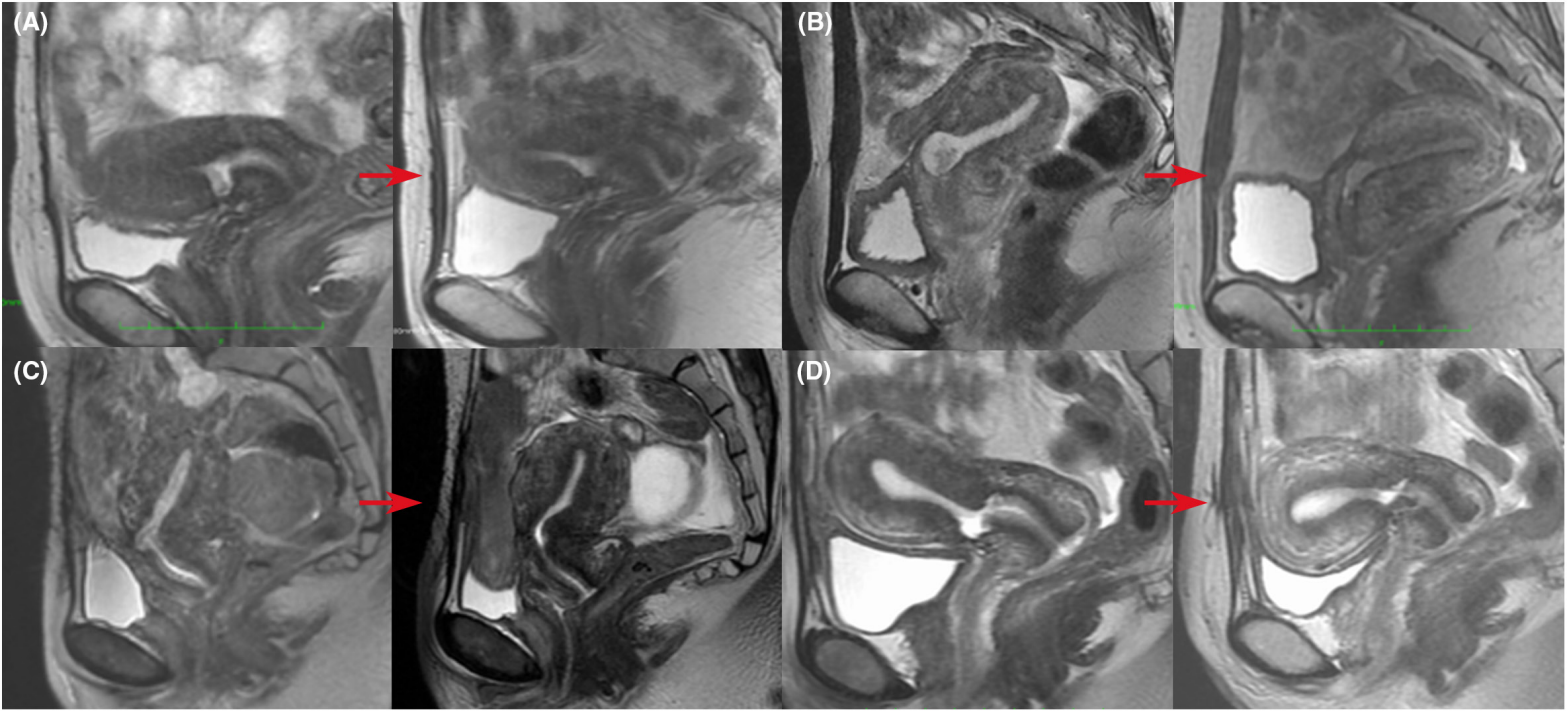
Magnetic resonance imaging findings before and after hysteroscopic surgery. Hysteroscopic surgery was performed for infertile women with cesarean scar disorder (CSDi) in each of the four independent cases (A–D). In all cases, the residual myometrial thickness increased, and postoperative pregnancies were established in all cases. Red arrows indicate pre‐ and post‐operative changes.

Some reports have shown that RMT was increased by resectoscopic surgery, whereas others did not significantly influence RMT.^71^, ^98^, ^99^, ^100^, ^101^ This difference may be attributed to different procedures of hysteroscopic surgery. In our study, only the inferior edge of the CSD was resected, and the entire isthmus was cauterized using a ball electrode. Therefore, the RMT became thicker after the resectoscopic surgery (Figure 2). Additionally, RMT thickening is a predictor of pregnancy prognosis.^101^

Florio et al.^102^ reported that the effectiveness of hysteroscopic surgery was superior to hormonal therapy in women with CSDi. Furthermore, in a randomized controlled study, hysteroscopic surgery was proven to be effective in treating symptoms associated with CSD.^82^ Pregnancy rates after hysteroscopic surgery have been reported between 40 and 100%. Based on these findings, we believe that hysteroscopic surgery is minimally invasive, safe, and effective in women with CSDi.

The indications for hysteroscopic surgery are constantly debated. Donnez and Vervoort selected the laparoscopic procedure when the RMT was <3 mm.^53^, ^82^ Tanimura et al. adopted this procedure when RMT was <2.5 mm. Cheng et al.^31^ adopted a criterion that the RMT should exceed 2 mm. These criteria were based on concerns regarding uterine perforation due to thin myometrium. Therefore, they were not based on clinical outcomes. In terms of effectiveness, Zeller et al.^103^ demonstrated no significant difference in postoperative pregnancy rate between severe defect (RMT ≤3 mm) and non‐severe defect (RMT >3 mm) groups. Although RMT alone could not identify indications for resectoscopic surgery, our group revealed the indication criteria for hysteroscopic surgery by adding age as a factor.^104^ The criterion was RMT ≥2.2 mm. Various infertility factors exist in older patients; therefore, it is difficult to determine the indications for surgery using RMT alone. However, infertility factors are limited to young patients. Therefore, analysis of non‐elder patients revealed that surgical indications can be determined using only CSD factors. Zhu Q also demonstrated that the key RMT was 2.2 mm in patients with AUB.^105^ According to a report by Zeller et al., there were two cases of perforation during surgery in the severe defect group.^106^ Therefore, when the RMT is <2.2 mm, we believe that laparoscopic surgery should be considered.

To date, there are no reports of uterine rupture during pregnancy following hysteroscopic surgery. If a patient undergoes a planned CS, we believe that there is a small risk of uterine rupture in a subsequent pregnancy after hysteroscopic surgery. Unlike laparoscopic surgery, hysteroscopic surgery does not involve anatomical repair. Therefore, uterine extension due to postoperative pregnancy may decrease RMT. If dehiscence is detected during CS, the trimming technique may have contributed to preventing the recurrence of CSDi.^107^ In this technique, the first layer is closed using a modified Gambee suture. This suture technique may contribute to a thicker RMT, even if a thinned myometrium is detected during the CS.

Endometriosis is often detected in the peritoneal cavity of women with CSDi. Therefore, we recommend that hysteroscopic surgery be performed under laparoscopy, particularly in cases of secondary infertility. When endometriosis was detected in the peritoneal cavity, it was simultaneously cauterized at the same time. Furthermore, even perforations can be repaired laparoscopically at the same time.

## 11 VAGINAL SURGERY IN WOMEN WITH CSDI

The first report of vaginal repair in women with CSDi was described by Klemm et al.,^108^ since then, there have been many reports, mainly from China. There are two arguments for suturing methods in vaginal surgery. The first is a single‐layer^108^, ^109^, ^110^, ^111^, ^112^, ^113^ or double‐layer^65^, ^80^, ^114^, ^115^, ^116^, ^117^, ^118^ closure. In the case of CS, the double‐layer procedure is superior to the single‐layer procedure in avoiding CSD. However, in the case of vaginal repair, there is no evidence indicating which method is better. Zhang et al. selected a single‐layer closure to reduce tissue ischemia.^111^ Second, the suture technique used was continuous or interrupted. However, almost all the suture techniques were interrupted (Table 3). Some authors have described that all secured knots are tightened after interrupted suturing to detect the appropriate point of insertion of the needle until the last suture.^108^, ^112^, ^114^

**TABLE 3:** Vaginal surgery for CSDi.

Author	Year	No.	Single or double layer	Interrupted or continuous	Pre‐RMT	Post‐RMT	Effectiveness	Pregnancy rate
Klemm et al	2005	5	Single layer	Continuous suture	NA	NA	AUB (100%)^a^	33%^b^
Khoshnow et al.	2010	1	Single layer	Interrupted suture	NA	NA	AUB (100%)	NA
Luo et al.	2012	42	Double layer	Interrupted suture	NA	NA	SR (93%)	NA
Chen Y et al.	2014	64	Single layer	Continuous suture	Range 2–5 mm	NA	SR (86%)	NA
Zhang X et al.	2016	14	Double layer	Interrupted suture	NA	NA	AUB (SI)	NA
Zhang Y et al.	2016	65	Double layer	Interrupted suture	NA	NA	AUB (89%)	NA
Zhou J et al.	2016	121	Double layer	Interrupted suture	2.6 ± 1.1 mm	8.65 ± 3.11 mm	AUB (SI)	NA
Zhou X et al.	2017	51	Double layer	Interrupted suture	2.7 ± 1.1 mm	5.7 mm (CSD existence)	AUB (SI)	NA
						9.2 mm (CSD disappearance)		NA
Chen H et al.	2019	100	Double layer	Interrupted suture	2.9 ± 1.1 mm (Anteflexion)	8.1 ± 2.6 mm (Anteflexion)	AUB (SI)	NA
		141	Double layer	Interrupted suture	2.5 ± 1.2 mm (Retroflexion)	7.5 ± 3.1 mm (Retroflexion)	AUB (SI)	NA
Wang Y et al.	2020	193	Double layer	Interrupted suture	2.5 ± 1.0 mm (1 previous C/S)	7.6 ± 3.0 mm (1 previous C/S)	AUB (SI)	NA
		55	Double layer	Interrupted suture	3.2 ± 1.6 mm (2 previous C/S)	8.2 ± 2.4 mm (2 previous C/S)	AUB (SI)	NA
Zhang YL et al.	2020	37	Single layer under SSL	NA	2.4 (2.0–4.5) mm	NA	AUB, QOL (SI)	49%
		37	Single layer	NA	2.6 (2.3–4.9) mm	NA	AUB, QOL (SI)	51%
Deng K et al.	2021	183	Single layer	Interrupted suture	2.3 ± 0.9 mm	5.3 ± 1.3 mm	AUB (SI)	70%
Mancuso et al.	2021	1	Single layer	Continuous suture	1.9 mm	NA	AUB (SI)	NA

Abbreviations: AUB, abnormal uterine bleeding; CSD, cesarean scar defect; CSDi, cesarean scar disorder; Dys, dysmenorrhea; Dysp, dyspareunia; IQR, interquartile range; NA, not available; NI, no improvement; No., the total number of cases in the study; QOL, quality of life; SI, significant improvement; SR, symptomatic relief; SSL, single‐site laparoendoscopic; (%), effective rate.

^a^ One patient was lost.

^b^ One patient did not wish for baby.

To date, all reports on vaginal repair have shown significant effectiveness for symptoms in women with CSDi (Table 3). Furthermore, some reports have demonstrated that vaginal repair contributes to an increased RMT. However, there is insufficient evidence regarding secondary infertility and subsequent pregnancy outcomes following vaginal repair of CSD. In a systematic review and meta‐analysis, Yuan et al.^119^ recently reported that hysteroscopic resection is superior to vaginal surgery in terms of blood loss, operative time, and hospital duration. However, there was no significant difference in the effectiveness of AUB and the restoration of RMT.

## 12 CONCLUSION

**FIGURE 3:**
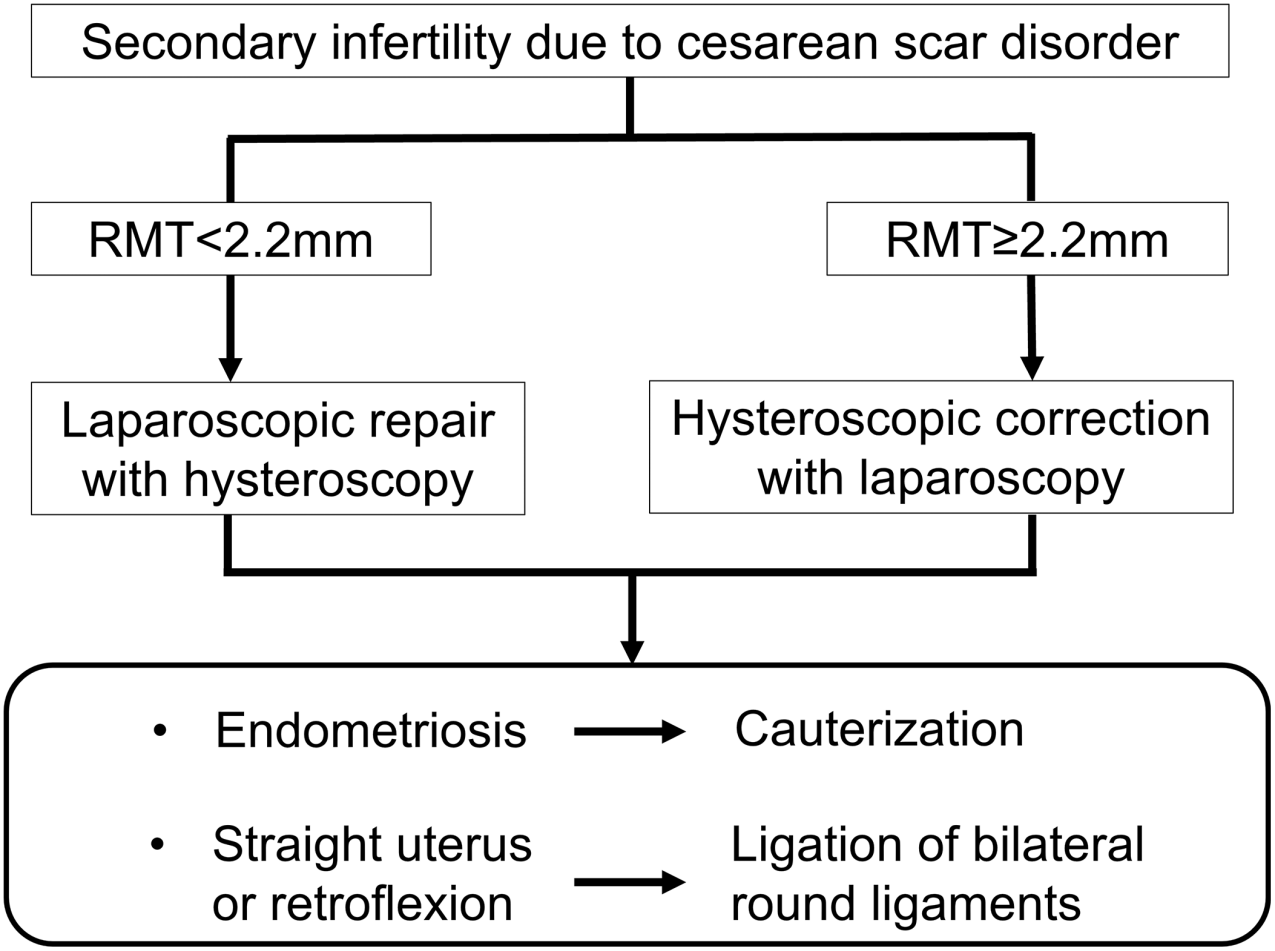
Treatment algorithm for secondary infertility in women with cesarean scar disorder. When residual myometrial thickness (RMT) is <2.2 mm, laparoscopic surgery with a hysteroscope is recommended because it is useful to accurately resect the cesarean scar defect (CSD). When RMT is ≥2.2 mm, hysteroscopic correction with laparoscopy is recommended because patients with cesarean scar disorder (CSDi) often have endometriosis. In both ways, the uterus is repositioned by suturing the round ligament to relieve tension on repaired CSD.

Cesarean scar defect was significantly associated with AUB and secondary infertility. Abnormal uterine bleeding is caused by the retention of menstrual blood and minor hemorrhage from the CSD. Minor infections may occur during fluid retention and chronic inflammation in the CSD and uterine cavity, eventually leading to decreased fertility. Because of these anatomical and physiological problems, it is difficult to conceive with conventional infertility treatments. Since there are no clear guidelines for surgical indications, the treatment policy must be determined based on the most recent evidence and the capabilities of the facility, whether drug treatment, general infertility treatment, ART or surgery, depending on the individual case. To relieve secondary infertility, many reports have suggested the effectiveness of laparoscopic repair and hysteroscopic correction; however, reports on the efficacy of vaginal surgery are insufficient compared to those of laparoscopic and hysteroscopic surgeries. In recent trends, patients with thin RMT have tended to avoid hysteroscopic surgery, which is generally less invasive than laparoscopic repair. Furthermore, we believe that laparoscopy should be used in combination with hysteroscopic surgery because endometriosis is often a complication in women with secondary infertility due to CSDi. Therefore, based on previous reports, we proposed a treatment algorithm for patients who wish to conceive (Figure 3).

So far, the lack of a definition for both CSD and symptoms due to CSD has led to variability in the frequency of CSD and clinical outcomes of several interventions for women with CSDi. We believe that conducting research using the same definition in the future will contribute to establishing more accurate frequency and treatment methods.

## FUNDING INFORMATION

Grants in Aid for Scientific Research (KAKENHI; 20K09616) supported this study and the preparation of this manuscript, including English language editing services.

## CONFLICT OF INTEREST STATEMENT

Takashi Murakami is an Editorial Board member of Reproductive Medicine and Biology and a co‐author of this article. To minimize bias, he was excluded from all editorial decision‐making related to the acceptance of this article for publication.

## HUMAN RIGHTS STATEMENT AND INFORMED CONSENT/ANIMAL STUDIES

This article does not contain any studies involving human and animal subjects performed by any of the authors.

## ETHICS STATEMENT

No ethical approval was needed for this review article.
